# Should transarterial chemoembolization be given before or after intensity-modulated radiotherapy to treat patients with hepatocellular carcinoma with portal vein tumor thrombus? a propensity score matching study

**DOI:** 10.18632/oncotarget.25224

**Published:** 2018-05-11

**Authors:** Xiaolong Li, Weixing Guo, Lei Guo, Wan Yee Lau, Naijian Ge, Kang Wang, Shuqun Cheng

**Affiliations:** ^1^ Eastern Hepatobiliary Surgery Hospital, The Second Military Medical University, Shanghai, China; ^2^ Department of Liver Surgery and Transplantation, Liver Cancer Institute and Zhongshan Hospital, Fudan University, Shanghai, China; ^3^ Faculty of Medicine, The Chinese University of Hong Kong, Shatin, Hong Kong SAR

**Keywords:** hepatocellular carcinoma, portal vein tumor thrombus, radiotherapy, transarterial chemoembolization

## Abstract

**Background and Objective:**

To compare the survival outcomes of patients with hepatocellular carcinoma (HCC) with portal vein tumor thrombus (PVTT) who received transarterial chemoembolization (TACE) before or after intensity-modulated radiotherapy (IMRT).

**Methods:**

During the study period, the survival outcomes of HCC patients with PVTT who underwent TACE before (TACE-RT) or after IMRT (RT-TACE) were compared. Using propensity score matching (PSM), matched pairs of patients were compared.

**Results:**

There were 76 patients in the TACE-RT group and 36 patients in the RT-TACE group. Using a 2:1 matching, 75 patients were included into this study after PSM: 50 patients in the TACE-RT group and 25 patients in the RT-TACE group. Before PSM, patients in the RT-TACE group showed significantly better survival when compared with the TACE-RT group (median survival, 13.2 months vs.7.4 months; *P* = 0.014) for patients with main trunk PVTT, and after PSM, the corresponding median survival was 13.2 months vs.7.4 months (*P* = 0.020). When compared with TACE-RT, RT-TACE had a significantly lower rate of worsening in liver function (9.5% vs. 33.3%, *P* = 0.044) for patients with main trunk PVTT.

**Conclusions:**

For HCC patients with main trunk PVTT, IMRT followed by TACE yielded better survival outcomes and liver function when compared to TACE followed by IMRT.

## INTRODUCTION

Hepatocellular carcinoma (HCC) is the fifth most common cancer and the second most common cause of cancer-related mortality [1]. Portal vein tumor thrombus (PVTT) is a poor prognostic factor of survival as it leads to portal hypertension, tumor spread, liver failure and death [2]. The Barcelona Clinic Liver Cancer (BCLC) group recommended sorafenib as the standard therapy for these patients [3, 4]. In Asian countries, liver resection has been proposed as an option that offers a chance of cure if the lesion is resectable [5, 6]. The optimal treatment for patients with HCC and PVTT remains controversial.

Recently, transarterial chemoembolization (TACE) has become the most popular palliative treatment for patients with unresectable HCC and is no longer considered as an absolute contraindication to patients with HCC with PVTT [7, 8]. Advanced radiotherapies (RT) including three-dimensional conformal radiotherapy (3D-CRT), stereotactic body radiotherapy (SBRT) and intensity-modulated radiotherapy (IMRT), have produced promising results in HCC patients with PVTT [9–11]. The combination of TACE and radiotherapy for HCC patients with PVTT significantly improved survival outcomes when compared with TACE or radiotherapy alone [12–19]. While most of these studies used TACE followed by RT, some applied RT before TACE [17]. In this study, the survival outcomes and adverse events of a cohort of HCC patients with different extent of PVTT underwent TACE either before or after IMRT were compared. In addition, a propensity score matching (PSM) analysis was done to minimize potential bias inherent to this retrospective, nonrandomized study. Independent prognostic factors associated with survival were also investigated.

## RESULTS

### Study population

**Figure 1:**
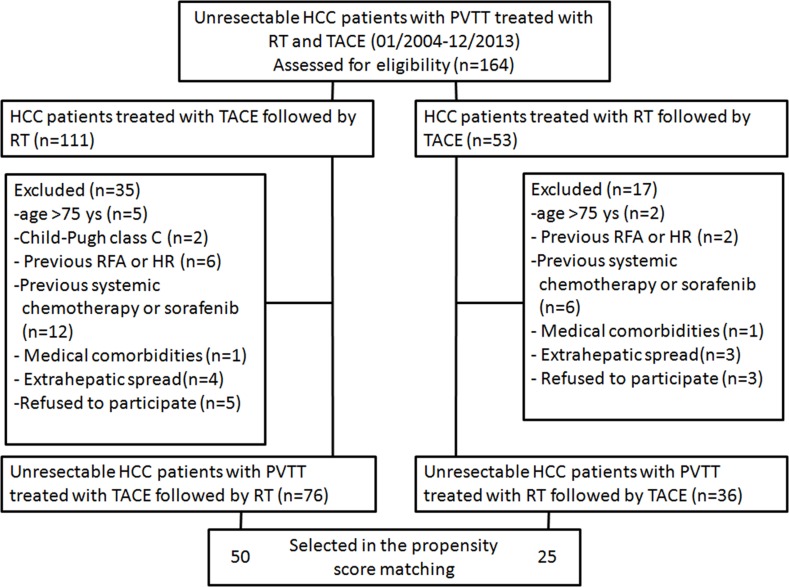
A flow chart of this study RFA = radiofrequency ablation; HR = hepatic resection.

Of 164 patients with unresectable HCC with PVTT who underwent combined TACE and IMRT during the study period, 52 patients were excluded because they met the exclusion criteria. Thus, 112 patients (the TACE-RT group, *n*=76; and the RT-TACE group, *n*=36) were included in this study. Of these, 75 patients with HCC with PVTT (the TACE-RT group, *n*=50; the RT-TACE group, *n*=25) were selected in the propensity score analysis (Figure 1).

The two groups showed no significant difference in all the baseline characteristics, which included age, sex, Child-Pugh score, total bilirubin (TBIL), albumin (ALB), alanine aminotransferase (ALT), HBsAg positivity, ascites, AFP level, tumor number, maximum lesion diameter, and extent of PVTT before and after propensity score matching (Table 1).

**Table 1:** Baseline characteristics of the two groups of patients before and after propensity score matching

Variable	Before propensity score matching			After propensity score matching		
	TACE-RT Group (*n*=76)	RT-TACE Group (*n*=36)	*P* value	TACE-RT Group (*n*=50)	RT-TACE Group (*n*=25)	*P* value
Sex Male Female	71 (93) 5 (7)	33 (92) 3 (8)	1.000	48 (96) 2 (4)	24 (96) 1 (4)	1.000
Age, yr ^a^	53.5 (45.3, 59.0)	52.0 (41.3, 60.8)	0.434	49.5 (42.5, 57.0)	46.0 (37.5, 56.0)	0.229
Child-Pugh score A B	73 (96) 3 (4)	32 (89) 4 (11)	0.296	48 (96) 2 (4)	24 (96) 1 (4)	1.000
Baseline laboratory test result ^a^ TBIL, μmol/L ALB, g/L ALT, U/L	17.0 (13.2, 23.0) 40.1 (36.1, 43.1) 46.0 (34.5, 65.7)	15.0 (13.0, 24.9) 38.1 (37.1, 40.2) 47.5 (26.3, 73.0)	0.348 0.065 0.739	17.5 (13.0, 23.3) 39.1 (35.1, 43.1) 45.0 (34.0, 77.8)	15.0 (12.5, 18.0) 38.1 (37.1, 41.4) 39.0 (25.5, 66.0)	0.314 0.595 0.202
HBsAg Positive Negative	67 (88) 9 (12)	32 (89) 4 (11)	1.000	49 (98) 1 (2)	24 (96) 1 (4)	1.000
HBV-DNA, copies/ml ≤ 10^4^ >10^4^	30 (45) 37 (55)	13 (41) 19 (59)	0.697	21 (43) 28 (57)	9 (37) 15 (63)	0.662
Ascites Absent Present	68 (90) 8 (10)	33 (92) 3 (8)	0.981	45 (90) 5 (10)	23 (92) 2 (8)	1.000
AFP, ng/ml ≤ 400 > 400	33 (43) 43 (57)	12 (33) 24 (67)	0.309	18 (36) 32 (64)	8 (32) 17 (68)	0.731
Tumor number 1 > 1	62 (82) 14 (18)	30 (83) 6 (17)	0.821	45 (90) 5 (10)	23 (92) 2 (8)	1.000
Maximum lesion diameter, cm ≤ 5 > 5	12 (16) 64 (84)	8 (22) 28 (78)	0.406	3 (6) 47 (94)	2 (8) 23 (92)	1.000
PVTT location nMT-PVTT MT-PVTT	40 (53) 36 (47)	15 (42) 21 (58)	0.278	20 (40) 30 (60)	10 (40) 15 (60)	1.000

^a^ Data are shown as median (interquartile range).

AFP, α-fetoprotein; ALB, Albumin; ALT, Alanine aminotransferase; HBsAg, Hepatitis B surface antigen; PVTT, Portal vein tumor thrombus; nMT-PVTT, non main trunk portal vein tumor thrombus; MT-PVTT, main trunk portal vein tumor thrombus; RT-TACE, radiotherapy followed by transarterial chemoembolization; TACE-RT, transarterial chemoembolization followed by radiotherapy; TBIL, Total bilirubin.

The median follow-up was 11 months (range, 2-48 months) for the TACE-RT group and 14 months (range, 2-55 months) for the RT-TACE group. Collectively, all of the patients in the RT-TACE and TACE-RT group died during the study period. Thirty-eight (50.0%) of the 76 patients in the TACE-RT group and 25 (69.4%) of the 36 patients in the RT-TACE group underwent repeated TACE, with a mean of 1.9 (range, 1–9) and 3.1 (range, 1–12) TACE procedures per patient in the two groups of patients, respectively.

### Tumor and PVTT response

The tumor and PVTT responses in patients with different extents of PVTT are shown in Table 2. For the liver tumors, the response rates for all patients were not significantly different between the TACE-RT and RT-TACE groups (59.2% vs. 72.2%, *P* = 0.182). On subgroup analysis, the response rate of the patients with main portal vein trunk tumor thrombus (main trunk-PVTT, MT-PVTT) in the RT-TACE group was 81.0%, which was significantly higher than the 50.0% in the TACE-RT group (*P* = 0.021). However, in patients without main portal vein trunk tumor thrombus (non-main trunk-PVTT, nMT-PVTT), the response rates between the two groups were not significantly different (67.5% in the TACE-RT group vs. 60.0% in the RT-TACE group, *P* = 0.602).

**Table 2:** Tumor and PVTT responses for patients with different extents of PVTT in the two treatment groups

		CR (n)	PR (n)	SD (n)	PD (n)	RR (%)	*P* value
***Tumor response***							
nMT-PVTT (*n*=55)	TACE-RT (*n*=40)	8	19	11	2	67.5	0.602
	RT-TACE (*n*=15)	2	7	5	1	60.0	
MT-PVTT (*n*=57)	TACE-RT (*n*=36)	2	16	14	4	50.0	0.021
	RT-TACE (*n*=21)	5	12	3	1	81.0	
All patients (*n*=112)	TACE-RT (*n*=76)	10	35	25	6	59.2	0.182
	RT-TACE (*n*=36)	7	19	8	2	72.2	
***PVTT response***							
nMT-PVTT (*n*=55)	TACE-RT (*n*=40)	8	21	9	2	72.5	0.928
	RT-TACE (*n*=15)	3	7	4	1	66.7	
MT-PVTT (*n*=57)	TACE-RT (*n*=36)	3	15	15	3	50.0	0.007
	RT-TACE (*n*=21)	6	12	2	1	85.7	
All patients (*n*=112)	TACE-RT (*n*=76)	11	36	24	5	61.8	0.094
	RT-TACE (*n*=36)	9	19	6	2	77.8	

^a^ Response rate (RR) was calculated with the following equation: RR = (CR+PR)/N.

^b^ CR is number of patients with complete response, PR is number of patients with partial response, SD is number of patients with stable disease, and PD is number of patients with progressive disease.

PVTT, Portal vein tumor thrombus; nMT-PVTT, non main trunk portal vein tumor thrombus; MT-PVTT, main trunk portal vein tumor thrombus; RT-TACE, radiotherapy followed by transarterial chemoembolization; TACE-RT, transarterial chemoembolization followed by radiotherapy.

For PVTT, the response rates of all patients were not significantly different between the TACE-RT and RT-TACE groups (61.8% vs. 77.8%, *P* = 0.094). On subgroup analysis, the response rate of patients with MT-PVTT in the RT-TACE group was 85.7%, which was significantly higher than the 50% rate in the TACE-RT group (*P* = 0.007). However, in patients with nMT-PVTT, the response rates between the two groups were not significantly different (72.5% in the TACE-RT group vs. 66.7% in the RT-TACE group, *P* = 0.928).

### Survival analysis of whole population with different extents of PVTT

**Figure 2:**
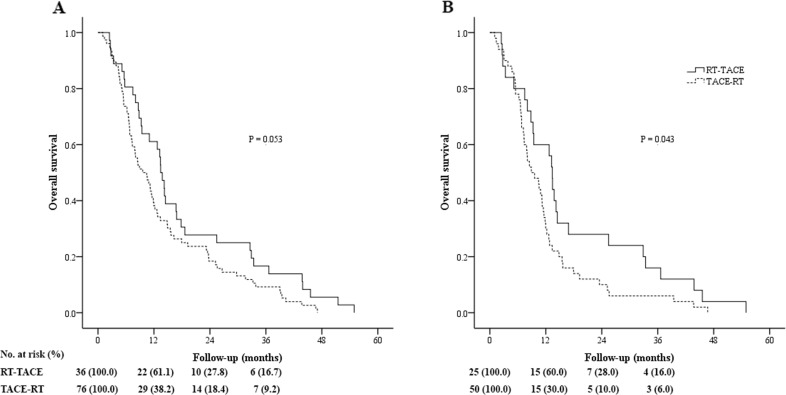
Kaplan-Meier curves of overall survival in patients with hepatocellular carcinoma (HCC) with portal vein tumor thrombus (PVTT) who underwent transarterial chemoembolization followed by radiotherapy (TACE-RT) or radiotherapy followed by transarterial chemoembolization (RT-TACE) **(A)** Whole study population (the TACE-RT group: n = 76, median OS = 9.6 months; the RT-TACE group: n = 36, median OS = 13.4 months; *P* = 0.053). **(B)** Patients selected in the propensity score analysis (the TACE-RT group: n = 50, median OS = 8.9 months; the RT-TACE group: n = 25, median OS = 13.4 months; *P* = 0.043).

**Figure 3:**
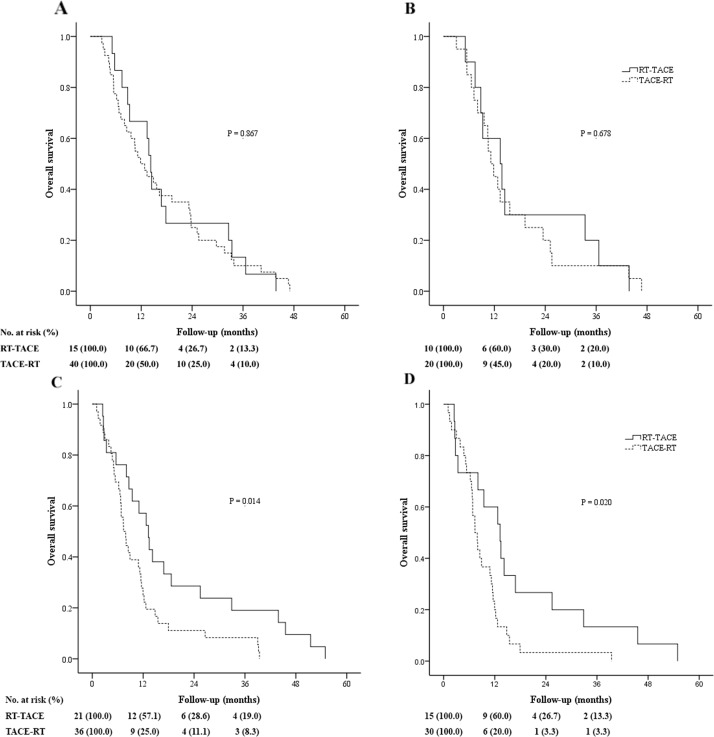
Kaplan-Meier curves of overall survival on subgroup analysis of patients with hepatocellular carcinoma (HCC) with different extents of portal vein tumor thrombus (PVTT) who underwent transarterial chemoembolization followed by radiotherapy (TACE-RT) or radiotherapy followed by transarterial chemoembolization (RT-TACE) **(A)** Whole study population of patients without main trunk PVTT (nMT-PVTT) (TACE-RT group: n = 40, median OS = 11.9 months; the RT-TACE group: n = 15, median OS = 14.2 months; *P* = 0.867). **(B)** Patients with nMT-PVTT selected in the propensity score analysis (the TACE-RT group: n = 20, median OS = 11.2 months; the RT-TACE group: n = 10, median OS = 13.4 months; *P* = 0.678). **(C)** Whole study population of patients with main trunk PVTT (MT-PVTT) (the TACE-RT group: n = 36, median OS = 7.4 months; the RT-TACE group: n = 21, median OS = 13.2 months; *P* = 0.014). **(D)** Patients with MT-PVTT selected in the propensity score analysis (TACE-RT group: n = 30, median OS = 7.4 months; RT-TACE group: n = 15, median OS = 13.2 months; *P* = 0.020).

The overall survival (OS) was similar between the TACE-RT and RT-TACE groups in all patients (*P* = 0.053; Figure 2A). The 1-, 2-, and 3-year OS rates were 38.2%, 18.4%, and 9.2% vs. 61.1%, 27.8%, and 16.7%, for the TACE-RT and RT-TACE groups, respectively. On subgroup analysis of OS in patients with different extents of PVTT, the TACE-RT group had similar OS as the RT-TACE group in patients with nMT-PVTT, (*P* = 0.867; Figure 3A). The 1-, 2-, and 3-year OS rates were 50.0%, 25.0%, and 10.0% vs. 66.7%, 26.7%, and 13.3%, for the TACE-RT and RT-TACE groups, respectively. In patients with MT-PVTT, the RT-TACE group had significantly better OS than the TACE-RT group (*P* = 0.014; Figure 3C). The 1-, 2-, and 3-year OS rates were 25.0%, 11.1%, and 8.3% vs. 57.1%, 28.6%, and 19.0%, for the TACE-RT and RT-TACE groups, respectively.

### Survival analysis of patients selected in the propensity score analysis with different extents of PVTT

For all patients selected in propensity score matching, the RT-TACE group had better OS than that of the TACE-RT group (*P* = 0.043; Figure 2B). The 1-, 2-, and 3-year OS rates were 30.0%, 10.0%, and 6.0% vs. 60.0%, 28.0%, and 16.0%, for the TACE-RT and RT-TACE groups, respectively. On subgroup analysis of OS in patients with different extents of PVTT, for patients with nMT-PVTT, the TACE-RT group had similar OS as the RT-TACE group (*P* = 0.678; Figure 3B). The 1-, 2-, and 3-year OS rates were 45.0%, 20.0%, and 10.0% vs. 60.0%, 30.0%, and 20.0%, for the TACE-RT and RT-TACE groups, respectively. In patients with MT-PVTT, the RT-TACE group had significantly better OS than the TACE-RT group (*P* = 0.020; Figure 3D). The 1-, 2-, and 3-year OS rates were 20.0%, 3.3%, and 3.3% vs. 60.0%, 26.7%, and 13.3%, for the TACE-RT and RT-TACE groups, respectively.

### Factors associated with overall survival

On univariable analysis for OS before PSM, maximum lesion diameter > 5 cm was associated with decreased long-term survival rates (*P* = 0.008, Table 3). In the Cox proportional hazards model, one independent prognostic predictor of poor survival was a maximum lesion diameter > 5 cm (HR: 2.004, 95% CI: 1.183-3.396, *P* =0.010).

**Table 3:** Univariable and multivariable analyses of survival for patients with hepatocellular carcinoma (HCC) with portal vein tumor thrombus (PVTT) before and after propensity score matching

		Univariable analysis		Multivariable analysis		
	*N*	Median OS (mo)	*P* value	HR	95% CI	*P* value
***All patients (n = 112)***						
Sex (male/female)	104/8	11.5/5.6	0.065			
Age (>60/≤60 yrs)	25/87	13.8/10.4	0.257			
Child-Pugh class (A/B)	105/7	11.2/8.6	0.781			
TBIL (≤34/>34 μmol/L)	102/10	11.5/5.2	0.480			
ALB (>35/≤35 g/L)	103/9	11.2/14.2	0.646			
ALT (≤40/>40 U/L)	45/67	12.7/10.6	0.132			
HBsAg (negative /positive)	13/99	14.9/11.0	0.080			
Ascites (absent/present)	101/11	11.2/8.6	0.370			
AFP (≤400/>400 ng/ml)	45/67	12.2/11.0	0.133			
Tumor number (1/>1)	92/20	10.9/14.8	0.074			
Maximum lesion diameter (≤5/>5 cm)	20/92	16.3/10.4	0.008	2.004	1.183-3.396	0.010
Main trunk PVTT (absent/present)	55/57	13.4/8.9	0.301			
Treatment (RT-TACE/TACE-RT)	36/76	13.4/9.6	0.053			
***Patients selected in the propensity score analysis (n = 75)***						
Sex (male/female)	72/3	10.6/7.9	0.230			
Age (>60/≤60 yrs)	10/65	10.9/10.4	0.677			
Child-Pugh class (A/B)	72/3	10.6/5.2	0.137			
TBIL (≤34/>34 μmol/L)	70/5	10.6/9.4	0.154			
ALB (>35/≤35 g/L)	69/6	10.6/7.4	0.658			
ALT (≤40/>40 U/L)	32/43	11.9/8.9	0.035	0.737	0.445-1.221	0.236
HBsAg (negative /positive)	2/73	8.1/10.9	0.441			
Ascites (absent/present)	68/7	10.6/9.6	0.964			
AFP (≤400/>400 ng/ml)	26/49	11.5/9.6	0.210			
Tumor number (1/>1)	68/7	10.9/8.0	0.126			
Maximum lesion diameter (≤5/>5 cm)	5/70	15.5/9.4	0.026	2.444	0.849-7.037	0.098
Main trunk PVTT (absent/present)	30/45	11.9/8.5	0.122			
Treatment (RT-TACE/TACE-RT)	25/50	13.4/8.9	0.043	0.655	0.396-1.082	0.098

95% CI, 95% confidence interval; AFP, α-fetoprotein; ALB, Albumin; ALT, Alanine aminotransferase; HBsAg, Hepatitis B surface antigen; HR, Hazard radio; OS, Overall survival; PVTT, Portal vein tumor thrombus; RT-TACE, radiotherapy followed by transarterial chemoembolization; TACE-RT, transarterial chemoembolization followed by radiotherapy; TBIL, Total bilirubin.

On univariable analysis of OS for patients after PSM, maximum lesion diameter > 5 cm, TACE-RT treatment, and ALT > 40 U/L were associated with a decreased survival (all *P* < 0.05). In the Cox proportional hazards model, no independent prognostic predictor was identified (Table 3).

### Adverse events

Table 4 shows the adverse events profile. During the period and between 1 and 3 months after treatment, worsening of liver function was observed in 22.4% (17/76) and 11.1% (4/36) of the patients in the TACE-RT and RT-TACE groups. The rate of liver function worsening in the TACE-RT group was higher when compared with the RT-TACE group, but there was no significant difference between the two groups (*P* = 0.154). On subgroup analysis of adverse events in the patients with different extents of PVTT, for patients with nMT-PVTT, the rates of liver function worsening were similar between the two groups (12.5% vs. 13.3%, *P* = 1.000). For patients with MT-PVTT, the rate of liver function worsening in the TACE-RT group was significantly higher than the RT-TACE group (33.3% vs. 9.5%, *P* = 0.044). Fever, gastrointestinal disorders, and acute bone marrow suppression were observed in 14.5% (11/76), 25.0% (19/76), and 25.0% (19/76), respectively, in patients in the TACE-RT group, and 13.9% (5/36), 22.2% (8/36), and 27.7% (10/36), respectively, in patients in the RT-TACE group. There were no significant differences between the two groups for these adverse events (*P* = 0.934, 0.748, and 0.754, respectively). All the disorders returned to baseline values within 3 weeks. No patients required discontinuation in treatment because of serious adverse reactions.

**Table 4:** Adverse events related to the two treatments in the different subgroups

	Worsening of liver function				Fever	Gastrointestinal disorders	Acute bone marrow suppression
	A to B	B to C	A to C	Sum			
***nMT-PVTT***							
TACE-RT (*n*=40)	4	1	-	5 (12.5%)	6 (15.0%)	10 (25.0%)	9 (22.5%)
RT-TACE (*n*=15)	2	-	-	2 (13.3%)	2 (13.3%)	3 (20.0%)	4 (26.7%)
*P* value				1.000	1.000	0.974	1.000
***mPVTT***							
TACE-RT (*n*=36)	10	1	1	12 (33.3%)	5 (13.9%)	9 (25.0%)	10 (27.8%)
RT-TACE (*n*=21)	2	-	-	2 (9.5%)	3 (14.3%)	5 (23.8%)	6 (28.6%)
*P* value				0.044	1.000	0.920	0.949
***All Patients***							
TACE-RT (*n*=76)	14	2	1	17 (22.4%)	11 (14.5%)	19 (25.0%)	19 (25.0%)
RT-TACE (*n*=36)	4	-	-	4 (11.1%)	5 (13.9%)	8 (22.2%)	10 (27.7%)
*P* value				0.154	0.934	0.748	0.754

nMT-PVTT, non-main trunk portal vein tumor thrombus; MT-PVTT, main trunk portal vein tumor thrombus; RT-TACE, radiotherapy followed by transarterial chemoembolization; TACE-RT, transarterial chemoembolization followed by radiotherapy.

## DISCUSSION

Accumulating evidences suggest that combined of TACE and RT is safe and effective to treat patients with unresctable HCC with PVTT [12–19]. Unfortunately, there are little data to suggest whether TACE should be given before or after external radiotherapy. Kang J et al [17] reported that there were no significant differences in response rate, survival rate, and α-fetoprotein level normalization rate between the SBRT-TACE group and the TACE-SBRT group. However, the latter regimen exerted a more severe negative effect on liver function when compared with the former one. For the whole population, our study showed similar results as reported by Kang J et al. On subgroup analysis using the extent of PVTT, for the first time our study showed that IMRT followed by TACE provided better survival outcomes and lower rates of worsening of liver function when compared with TACE followed by IMRT in HCC patients with main trunk PVTT. Using propensity score matching analysis which reduced selection biases and increased reliability of the study, this study also substantiated the above observations.

When PVTT extends to the main portal vein, the prognosis is extremely bad because (1) shedding of HCC cells leads to extensive intra-hepatic metastases; (2) complete obstruction of the main portal vein causes further deterioration in liver function. In a recent review article on the role of radiotherapy as a treatment modality for patients with HCC and PVTT, main PVTT serves as a main obstacle to liver function maintenance and a source of metastasis, and IMRT can be an effective treatment for main PVTT [20]; (3) aggravation of portal hypertension results in intractable ascites and esophageal bleeding [21]. Many HCC patients with main trunk PVTT die of esophageal bleeding or liver failure caused by obstruction of the main portal vein, rather than from the liver tumor. The placement of a portal vein stent in patients with HCC with main PVTT to keep the main portal vein patent has been shown to play a prominent role in improving hepatic function, in preventing liver failure and variceal bleeding due to portal hypertension [22]. Thus, timely radiotherapy to control PVTT may be beneficial for these patients.

For patients with main trunk PVTT, TACE before IMRT can lead to hepatic ischemia and cellular damage, which can worsen liver function, reduce some effects of IMRT as tumor hypoxia caused by TACE, and enhance radioresistance in HCC [23]. On the other hand, IMRT before TACE can shrink the PVTT. Subsequently increased blood flow through the main portal vein can reduce the negative embolic effects of TACE, and the worsening effects on liver function caused by the combined treatment. Liver function is recognized to be an independent prognostic factor of survival of HCC patients with PVTT [24, 25]. These may help to explain why IMRT followed by TACE resulted in less worsening of liver function and significantly better survival outcomes for HCC patients with main trunk PVTT when compared with TACE followed by IMRT.

Our study had limitations. First, this is a retrospective study carried out in a single-center which have potential selection biases. However, we attempted to use propensity score matching analysis to overcome this limitation. Second, the aetiology of patients with HCC and PVTT in our study is mainly due to chronic hepatitis B. The results of this study may not be applied to HCC due to other aetiologies. Finally, a prospective randomized trial with considerable population is necessary to confirm our findings.

In conclusion, for HCC patients with main trunk PVTT, IMRT followed by TACE produced better survival outcomes and caused less worsening of liver function than TACE followed by IMRT.

## MATERIALS AND METHODS

### Patients

The study was approved by the Ethics Committee of the Eastern Hepatobiliary Surgery Hospital of the Second Military Medical University (Shanghai, China). Written informed consent was obtained from all the patients for their data to be used for clinical research.

Between January 2004 and December 2013, all consecutive patients with unresectable HCC with PVTT who were treated by combined TACE and IMRT at the Eastern Hepatobiliary Surgery Hospital of the Second Military Medical University (Shanghai, China) were retrospectively studied. The inclusion criteria were: (a) age between 18 and 75 years, (b) HCC with major portal vein invasion, (c) the tumor was considered to be unresectable by two senior hepatic surgeons, (d) Child-Pugh A or B liver function. The exclusion criteria were: (a) Child-Pugh C liver function, (b) extra-hepatic metastasis, (c) previously treated by hepatic resection (HR), radiofrequency ablation (RFA), sorafenib or systemic chemotherapy, (d) serious associated medical diseases.

HCC was diagnosed according to the non-invasive criteria of the European Association for the Study of Liver/American Association Guidelines [26]. The presence and extent of PVTT were assessed on multiphase dynamic CT scans using the following criteria: a low-attenuation intraluminal mass which expanded the portal vein or as filling defects in the main portal vein trunk, portal venous branches, or both. The patients were divided into two subgroups according to whether the tumor thrombus had extended to the main portal vein trunk: Subgroup 1, extent of PVTT not involving the main portal vein (Cheng's classification I and II; Japanese Classification vP1 to vP3); and Subgroup 2, PVTT had extended to the main portal vein (Cheng's classification III and IV; Japanese Classification vP4) [27].

The treatment decision was made by our multi-disciplinary team that the patients were suitable for treatment with either TACE before (TACE-RT) or after IMRT (RT-TACE). Patients were considered to be suitable for TACE only if they had good liver function and there were adequate venous collaterals around the blocked main portal veins [28, 29]. The final decision on the treatment was made by the patient after full explanation was given to the patient.

### The TACE procedure

Using the Seldinger technique, a vascular catheter was inserted through a femoral artery to the hepatic artery. After hepatic angiography, the catheter was selectively inserted into the tumor-feeding artery whenever technically possible. An emulsion of 5-fluorouracil (1 g), mitomycin C (20 mg), cisplatin (5 mg), and lipiodol 10 to 30 ml (1 to 2 ml/cm diameter of the tumor) was injected. Gelfoam fragments were then injected to embolise the tumor-feeding vessel. The treatment was repeated at 6 to 8 weeks intervals until complete disappearance of viable intrahepatic tumor, provided that hepatic function was preserved.

### Radiotherapy procedure

IMRT was used for radiotherapy. The patient was scanned during the arterial phase and portal phase, from carina to the fifth lumbar vertebra with a thickness slice of 5.0 mm. The images and related data were delivered to the treatment plan system (TPS). The PVTT was outlined as a clinical target volume (CTV), and the plan target volume (PTV) was expanded 1.0 cm in the XY axis, and 0.5 cm in the Z axis (head direction). The prescribed doses to the initial PTV ranged from 50 to 67 Gy (median 58 Gy), given in daily doses of 2.0-2.2 Gy. The biologically effective dose (BED) ranged from 61.0 Gy to 82.5 Gy (median 68.2 Gy, α/β=10). A dose volume histogram (DVH) was used for dose optimization, with 90% of the dose curve being completely covered by the PTV. The internal dose to the PVTT was uniform, and the dose change was never more than 5%. The radiation doses to the organs such as the gastrointestinal tract and the spinal cord were all acceptably low and the dose limit of a high dose was never more than 10%.

### Combination treatment process

All patients received treatment within 1 week of diagnosis of HCC with PVTT. The group assignment was: for group TACE-RT, 76 patients were treated with TACE followed by IMRT within the next 2-4 weeks, and for group RT-TACE, 36 patients were treated with IMRT followed by TACE within the next 2-4 weeks, depending on recovery of liver function. Patients with a high viral load (≥ 10^4^ copies/ml) of HBV-DNA were given nucleotide/nucleoside analog (NA) treatment [30].

### Evaluation

All patients were followed-up by the same multidisciplinary team after treatment. This study was censored on June 30, 2017. The baseline characteristics of the two groups were compared before and after propensity score matched analysis. The short-term therapeutic effects on liver tumors and PVTT were evaluated separately according to the modified Response Evaluation Criteria in Solid Tumors (mRECIST) [31, 32] within 1 month after treatment. A complete response (CR) was defined as disappearance of intratumoral arterial enhancement in all target lesions; a partial response (PR) was at least 30% decrease in the sum of diameters of viable (contrast enhancement in the arterial phase) target lesions, taking as reference the baseline sum of the diameters of the target lesions; a progressive disease (PD) was an increase of at least 20% in the sum of the diameters of viable (enhancing) target lesions, taking as reference the smallest sum of the diameters of viable (enhancing) target lesions recorded since treatment started; a stable disease (SD) was any cases that do not qualify for either partial response or progressive disease. The response rate (RR) was estimated based on the combined number of patients with CR and PR. In addition to the size of the PVTT, other factors like recanalization of any part of the blocked portal vein were also taken into consideration.

For long-term survival outcomes, OS was compared between the two groups before and after propensity score matched analysis. OS was measured from the date of first treatment (either TACE or IMRT) to the date of death or the last follow-up. The prognostic factors for overall survival were explored using univariable and multivariable analyses before and after propensity score matched analysis. A subgroup analysis was performed on patients with different extents of PVTT.

The adverse events were evaluated according to the Common Terminology Criteria for Adverse Events (CTCAE; version3.0) [33].

### Statistical methods

All statistical analyses were performed using SPSS 20.0 (IBM, New York, NY). Continuous variables were expressed as mean ± sd or median (interquartile range) as appropriate. Categorical variables were compared by the χ^2^ test or the Fisher exact test, and continuous variables by the student's t test or the non-parametric test. Overall survival curves were constructed using the Kaplan-Meier method and compared with the log-rank test. Univariable analyses were performed with the log-rank test. Variables with a *P* value of < 0.05 were then entered into a multivariable analysis. Multivariable analyses were performed with a Cox proportional hazard regression model.

To investigate the association between treatment and outcomes in an observational database rather than in a randomized, controlled trial, a propensity score matching analysis was used in an attempt to reduce biases in patient selection. The R version 2.12.1 (The R Foundation for Statistical Computing) and the MatchIt package were used to produce the propensity score graphs. PSM was performed via binary logistic regression to generate a propensity score for each patient. The co-variables entered into the model included age, sex, Child-Pugh score, HBsAg positivity, AFP, PVTT extent, tumor size, and tumor number. Subsequently, a one-to-two match between the RT-TACE group and the TACE-RT group on patients with HCC with PVTT was obtained by use of the nearest-neighborhood matching using a caliper width of 0.02 without replacement.

All statistical tests were two tailed, and a *P* value < 0.05 indicated a significant difference.
